# Joint Preservation of the Wrist Using Articulated Distraction Arthroplasty: A Case Report of a Novel Technique

**DOI:** 10.1155/2015/812807

**Published:** 2015-02-12

**Authors:** Matt D. A. Fletcher

**Affiliations:** ^1^University of British Columbia, West Mall, Vancouver, BC, Canada V6T 1Z4; ^2^Dawson Creek & District Hospital, 11100-13th Street, Dawson Creek, BC, Canada V1G 3W8

## Abstract

Distraction arthroplasty of the ankle, elbow, and hip has become widely accepted and used within the orthopaedic community with excellent initial results which appear sustained. To date it has not been applied to the wrist in the same manner. A novel technique, drawn upon past success of articulated ankle distraction and static wrist distraction, was devised and evaluated by application of articulated wrist distraction performed over a 12-week period in a patient with poor functional outcome following limited wrist fusion. Posttreatment results showed improvement in range of motion (100-degree arc), subjective pain, and functional outcome measures (DASH 21.7, Mayo Wrist Score 80) comparable or better than either limited wrist fusion or proximal row carpectomy. Articulated wrist distraction initially appears to be a promising therapeutic option for the management of the stiff and painful wrist to maintain maximal function for which formal wrist arthrodesis may be the only alternative.

## 1. Introduction

Articulated joint distraction was first described in the knee and elbow in 1975 by Volkov and Oganesian [1]. This technique has been further developed and applied to other large joints and has subsequently been described as distraction arthroplasty due to the positive symptomatic benefits in osteoarthritic joints, with corresponding improved function [2]. Distraction arthroplasty of the elbow, knee, ankle, and hip has been shown to be highly beneficial in the management of osteoarthritis and posttraumatic stiffness [1–5]. Key results have been the improvement of range of motion, relief of pain, and improvement in radiological appearance of the joint in terms of reduction of sclerosis and increase in joint space [5–7]. It is postulated that the distraction technique results in histiogenesis not only of the integument and periarticular structures but also within the joint itself with the generation of cartilage homologue tissue [5].

Distraction of the wrist has not been investigated to the same extent and has been previously focussed on the correction of stiffness [8] with less attention to global functional improvement of the joint through active range of motion and function during distraction. Drawing on the success of ankle joint distraction arthroplasty, a technique was modified to apply the same principles to the wrist for joint preservation in an individual in whom wrist fusion would have had severe consequences. The key feature of this technique was to permit and encourage active usage and range of motion during distraction to apply the principles of distraction arthroplasty in the joint rather than pure correction of range of motion.

## 2. Case Presentation

The technique of articulated distraction was applied to the wrist by applying the concepts learnt in distraction arthroplasty of the ankle [5] and developing an articulated frame construct which could be utilised in the wrist. This was tested as follows.

**Figure 1:**
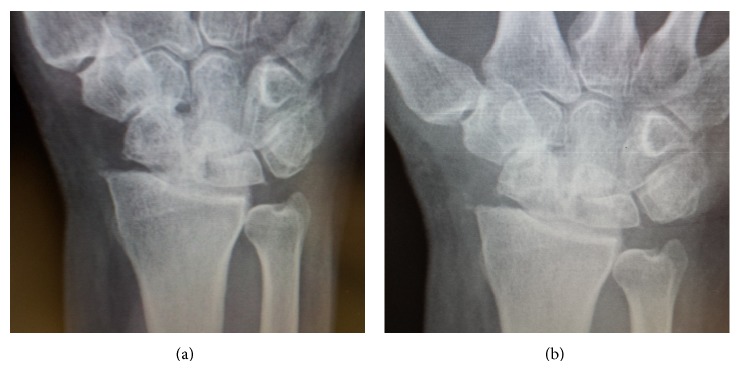
Preoperative appearance of wrist showing radiocarpal osteoarthritis.

A 58-year-old paramedic presented with a stiff painful wrist following limited wrist fusion (LWF) with scaphoid retention (scaphocapitolunate fusion) and styloidectomy for a neglected scapholunate disassociation with stage 3 scapholunate advanced collapse (SLAC) and subsequent removal of hardware and open capsular release with significant radiocarpal osteoarthritis (Figure 1). Due to occupational and recreational demands of active wrist range of motion and strength (performance of prolonged cardiopulmonary resuscitation and the use of a motorcycle throttle) formal wrist arthrodesis was contraindicated and the patient had previously refused proximal row carpectomy (PRC). Preoperative range of motion was 0 degrees dorsiflexion to 25 degrees palmar flexion, DASH score was 69.2, and the Mayo Wrist Score (MWS) was 15. Pain Visual Analogue Scale was 3-4 at rest and 7–9 on manual activity. The technique of experimental articulated distraction as salvage with an attempt to avoid fusion was proposed and informed consent was given by the patient.

**Figure 2:**
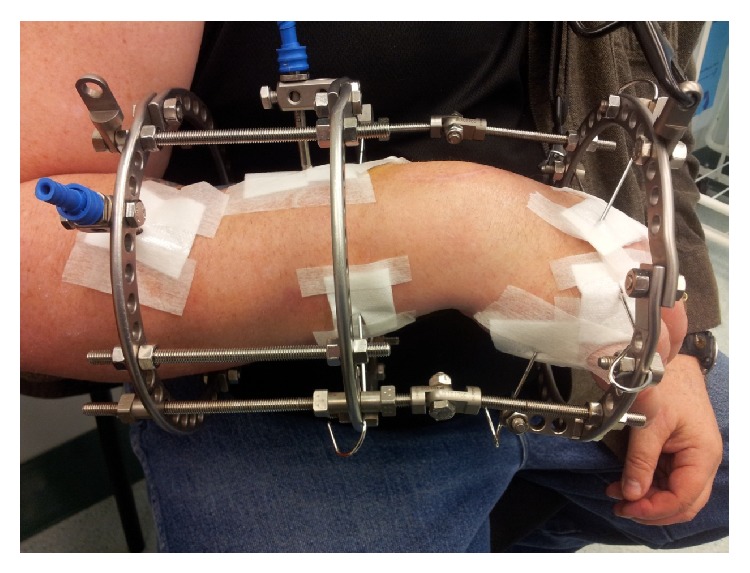
Ilizarov wrist distraction fixator assembly.

A circular Ilizarov fixator was assembled and applied over the wrist and hand with a transfixion wire traversing the distal metaphyseal radius and ulna orthogonal to the plane of the forearm in neutral position and a more proximal dorsoradial Schanz screw, with crossed olive wires through the distal metacarpus. Transfixion elements were inserted according to accepted zones to avoid iatrogenic nerve injury [8]. Intraoperative screening confirmed that the limited wrist fusion was solid and that no motion occurred through the midcarpal row. Although the wrist joint is a structure with complex kinematics, the hinges were located on the transstyloid axis due to the arthritis having occurred maximally in the radiocarpal joint. A 2 mm distraction was immediately applied across the radiocarpal joint (Figure 2).

**Figure 3:**
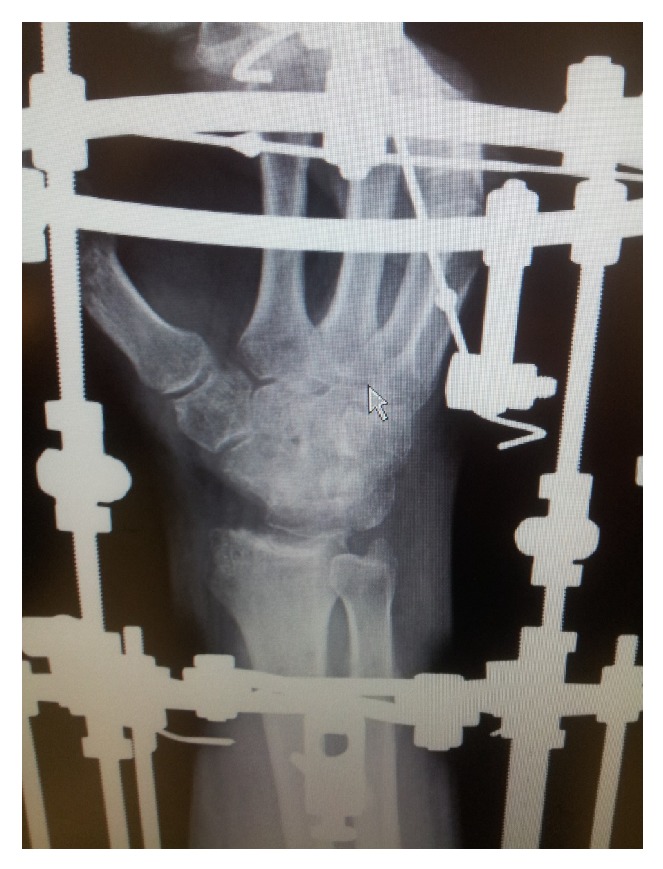
Maximal wrist distraction in frame.

A further 5 mm distraction was applied over 12 days paying careful attention to neurological function (Figure 3). During an initial distraction rate of 1 mm per day, mild median nerve sensory symptoms were noted by the patient; thus distraction was slowed with full resolution of mild paraesthesia. At this point, full range of motion and use were allowed within the parameters of the frame. Due to hinge position, the carpus was prevented from translation or rotation whilst carrying out flexion and extension. Note was made during this time of the tendency of the wrist to enter ulnar deviation and thus the distraction was increased on the ulnar aspect of the frame to correct this. Outriggers were attached to the frame to allow progressive passive correction of range of motion, performed by the patient daily, with 50-degree dorsiflexion and 45-degree palmar flexion achieved through the frame limited by frame impingement. A standard pin site care protocol of daily cleaning with sterile saline and occlusive dressing was used; there was one minor pin tract infection during frame treatment that was successfully treated with antibiotics. The frame was removed at 12 weeks following application and physiotherapy commenced.

**Figure 4:**
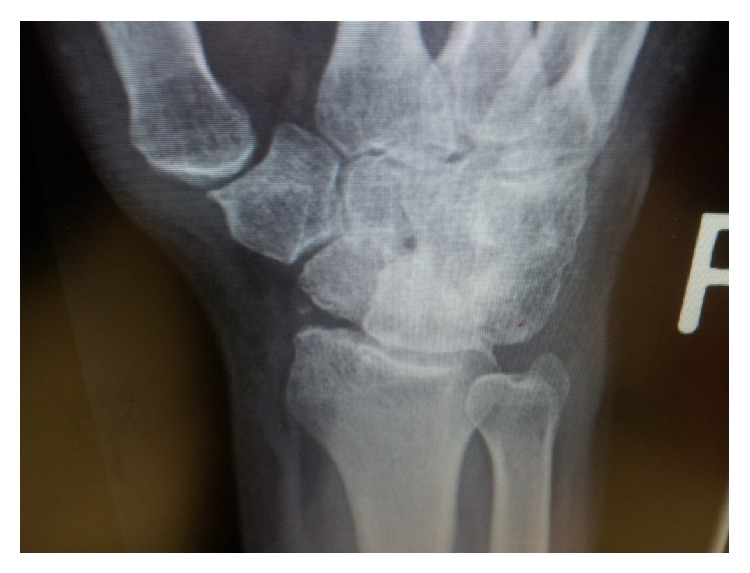
Postdistraction radiograph showing improved radiocarpal appearance.

**Figure 5:**
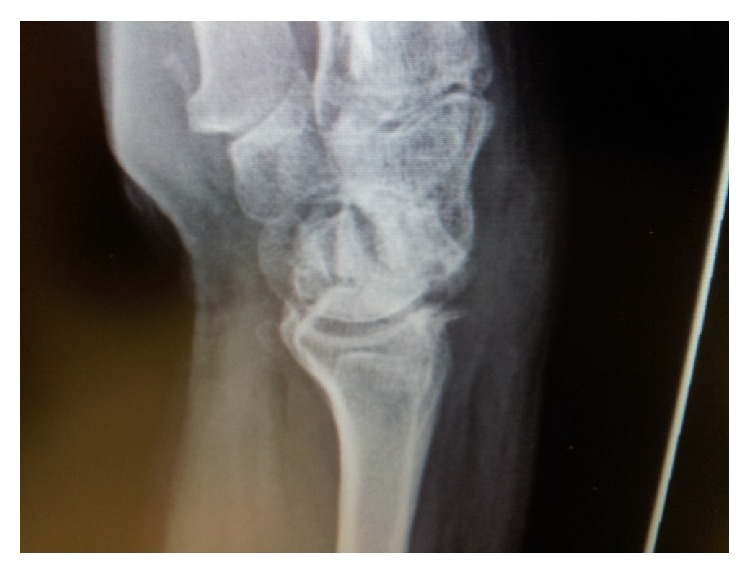
Postdistraction radiograph showing improved radiocarpal joint line.

**Figure 6:**
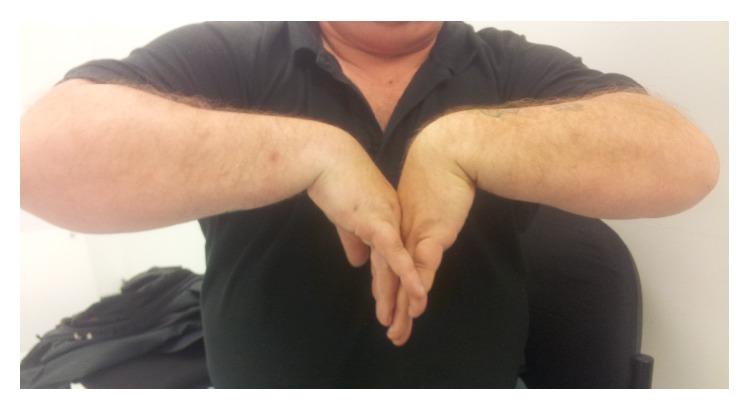
Postdistraction wrist flexion.

**Figure 7:**
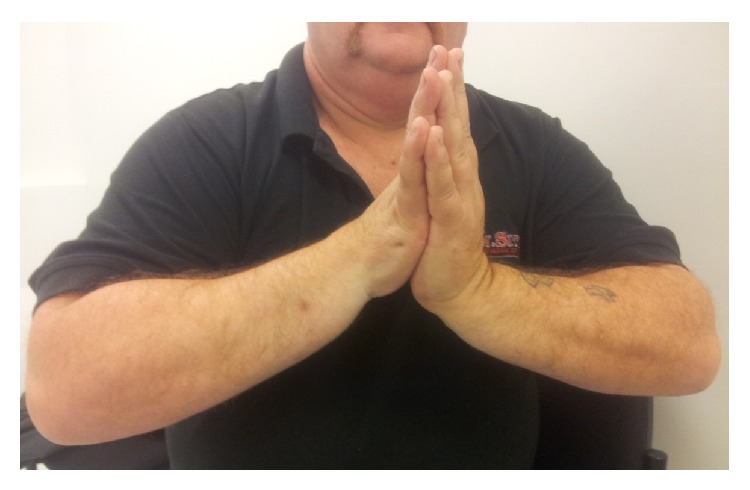
Postdistraction wrist extension.

**Figure 8:**
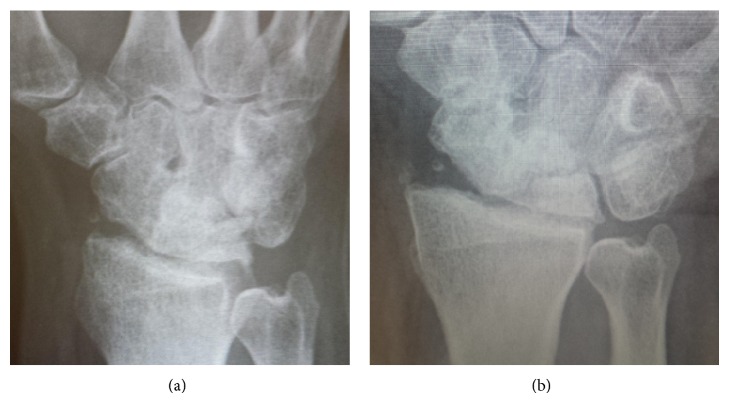
Wrist radiographs showing maintained joint space at 2 years.

At 20 weeks following frame removal final clinical range of motion was from 30-degree dorsiflexion to 70-degree palmar flexion with a 30-degree arc range of motion in radioulnar deviation and pain was significantly improved (Figures 4, 5, 6, and 7). Grip strength was subjectively rated as good. Radiologically, increased joint space of 1.5 mm was demonstrated. All measurements of radiocarpal joint space were performed utilising calibrated digital radiography with a standardised magnification factor, measuring between the scaphoid fossa and the scaphoid. This was maintained at 12- and 24-month review without recurrence of symptoms or significant sclerosis (Figure 8). He was able to return to full duties of his occupation without restriction. MWS had improved to 80 and DASH to 21.7 and pain was subjectively much improved with a VAS of 1 at rest and 3-4 on manual activity.

## 3. Discussion

Radiocarpal osteoarthritis, scapholunate advanced collapse, and posttraumatic wrist stiffness are significant problems which carry major functional morbidity and symptomatology. To date LWF with or without scaphoid excision or PRC affords maximal functionality and formal wrist arthrodesis maximal pain relief at the expense of mobility [9, 10]. Many patients are unwilling to contemplate further loss of range of motion in the form of radiocarpal arthrodesis, and PRC is not without its opponents with a 12–30% failure rate and conversion to arthrodesis at 5.5 years, definite reduction in grip strength to between 50 and 90% of the contralateral wrist, and up to 75% patient dissatisfaction [10–14]. The patient described here had a poor outcome following LWF and refused PRC or formal radiocarpal fusion.

Distraction of stiff soft tissues and scar tissue is well recognised to be of benefit and has found considerable success with elbow arthrofibrosis [3], postburn knee stiffness [14], and equinus deformity of the ankle [4] in particular. Over the past decade, the concept of distraction arthroplasty has been developed [5, 15]. This draws on the successes of distraction to improve range of motion but adds an additional component in the form of free articulation. This technique has shown very positive results in the management of degenerative disease of the ankle and in the management of paediatric hip pathologies [2, 16].

The technique of articulated distraction relies on slow distraction at 1 millimetre a day of a given joint to a maximum of 7–10 millimetres total joint distraction in a hinged fixator, followed by functional use and free range of motion for at least a 10–12-week period. Evidence from the literature supports the concept of distraction histiogenesis occurring with the joint, with the off-loading of sclerotic periarticular bone and subsequent improvement in the appearance of sclerosis, decrease in shear on remaining articular cartilage with opportunity for cartilage healing [17], improvement in range of motion, and improvement in pain, which appears to be sustained in the short to medium term [5, 18]. Permitting motion during distraction appears to convey sustained additional benefit [7, 16].

Wrist distraction has been described in the literature in limited series with success in improving range of motion of the stiff wrist [8]; however the focus of that intervention had been specifically to improve range of motion rather than to attempt global improvement of wrist symptoms and function.

Mechanical distraction of the ankle is typically performed to a 5–10 mm gap [5, 18]. In this wrist, a 7 mm distraction gap was utilised, based on evidence from the literature derived from external fixation distraction of the distal radial fracture which suggested that distraction of 3 mm fully tensions radiocarpal ligamentous structures [19]; that distraction of 5–8 mm is not associated with residual radiocarpal, intercarpal, or metacarpal stiffness [20]; and that greater than 8 mm distraction increases force to greater than 80 N through the carpus and thus may be excessive [21].

During distraction of the wrist in this case, a tendency towards palmar flexion and ulnar deviation deformity was noted, with very rapid improvement in maximal palmar flexion but considerably more difficulty achieving dorsiflexion which was addressed by the use of outriggers. This mirrors previous cadaveric findings of the effects of traction on wrist kinematics [22].

Final arc range of motion in this patient was 100 degrees, which compares very favorably to that seen in PRC of 81 degrees and LWF of 80 degrees, respectively [9]. Further study is necessary as to whether this range is maintained over time.

Radiocarpal arthrodesis remains a final and definitive technique for the management of the painful and degenerate wrist, regardless of aetiology [23]. Whilst pain is reliably improved, the absolute loss of range of motion precludes many activities and may be incompatible with the patient's occupation [24]. All other reasonable options should be excluded prior to such an intervention. Complication rates are high, and patient satisfaction is variable [23, 25]. Wrist arthroplasty remains in debate for all but patients with severe rheumatoid arthritis and lower functional demands and is not yet an option for patients with manual occupation or who require a combination of strength and range of motion [26].

Whilst this case is a preliminary report of functional distraction arthroplasty of the wrist as a salvage option prior to considering radiocarpal fusion and greater study of this technique needs to be performed, initial results from this case suggest that this may be a useful additional option in the armamentarium of the orthopaedic surgeon, particularly as it does not preclude future conversion to radiocarpal arthrodesis. The initial functional results appear to be far greater than those provided by formal arthrodesis.
